# 3-(3-Nitro­benz­yl)-4*H*-chromen-4-one

**DOI:** 10.1107/S1600536813003589

**Published:** 2013-02-09

**Authors:** Kaalin Gopaul, Neil Anthony Koorbanally, Mahidansha Shaikh, Hong Su, Deresh Ramjugernath

**Affiliations:** a School of Chemistry and Physics, University of KwaZulu-Natal, Private Bag X54001, Durban 4000, South Africa; b Chemistry Department, University of Cape Town, Rondebosch 7701, South Africa; c School of Chemical Engineering, University of KwaZulu-Natal, Durban 4041, South Africa

## Abstract

In the title compound, C_16_H_11_NO_4_, the dihedral angle between the 10-membered coplanar chromone ring system and the benzene ring is 77.83 (3)°. In the crystal, weak C—H⋯O hydrogen bonds link the mol­ecules into a three-dimensional network.

## Related literature

For the preparation, see: Valkonen *et al.* (2012 ▶). For related structures, see: Sievänen *et al.* (2010 ▶); Gopaul *et al.* (2012 ▶); Valkonen *et al.* (2012 ▶). For general background to homoisoflavoinoids, see: Shaikh *et al.* (2011 ▶); du Toit *et al.* (2010 ▶).
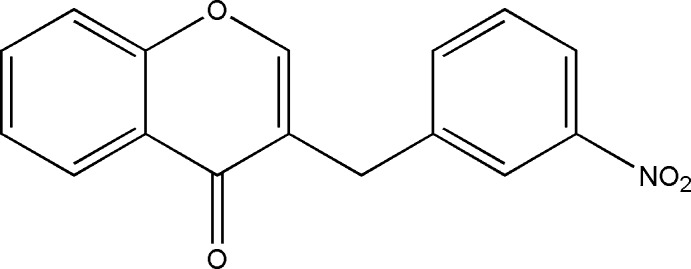


## Experimental

### 

#### Crystal data

C_16_H_11_NO_4_
*M* *_r_* = 281.26
Monoclinic, 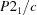

*a* = 4.6082 (3) Å
*b* = 10.4219 (6) Å
*c* = 26.4468 (17) Å
β = 90.428 (1)°
*V* = 1270.10 (14) Å^3^
*Z* = 4
Mo *K*α radiation
μ = 0.11 mm^−1^
*T* = 173 K
0.42 × 0.22 × 0.04 mm

#### Data collection

Bruker Kappa DUO APEXII diffractometer
Absorption correction: multi-scan (*SADABS*; Sheldrick, 1997 ▶) *T*_min_ = 0.956, *T*_max_ = 0.996
16973 measured reflections
4246 independent reflections
3069 reflections with *I* > 2σ(*I*)
*R*_int_ = 0.030

#### Refinement

*R*[*F* ^2^ > 2σ(*F* ^2^)] = 0.047
*wR*(*F* ^2^) = 0.138
*S* = 1.04
4246 reflections
190 parameters
H-atom parameters constrained
Δρ_max_ = 0.44 e Å^−3^
Δρ_min_ = −0.19 e Å^−3^

### 

Data collection: *APEX2* (Bruker, 2006 ▶); cell refinement: *SAINT* (Bruker, 2006 ▶); data reduction: *SAINT*; program(s) used to solve structure: *SHELXS97* (Sheldrick, 2008 ▶); program(s) used to refine structure: *SHELXL97* (Sheldrick, 2008 ▶); molecular graphics: *ORTEP-3* (Farrugia, 2012 ▶); software used to prepare material for publication: *SHELXL97*.

## Supplementary Material

Crystal structure: contains datablock(s) I, global. DOI: 10.1107/S1600536813003589/ff2097sup1.cif

Structure factors: contains datablock(s) I. DOI: 10.1107/S1600536813003589/ff2097Isup2.hkl

Supplementary material file. DOI: 10.1107/S1600536813003589/ff2097Isup3.cml

Additional supplementary materials:  crystallographic information; 3D view; checkCIF report

## Figures and Tables

**Table 1:** Hydrogen-bond geometry (Å, °)

*D*—H⋯*A*	*D*—H	H⋯*A*	*D*⋯*A*	*D*—H⋯*A*
C2—H2⋯O2	0.95	2.32	3.2663 (15)	171
C7—H7⋯O3^i^	0.95	2.65	3.5614 (19)	160
C8—H8⋯O3^ii^	0.95	2.54	3.3071 (18)	138
C14—H14⋯O4^iii^	0.95	2.50	3.4380 (17)	172
C15—H15⋯O1^iv^	0.95	2.62	3.5569 (15)	170
C16—H16⋯O2^v^	0.95	2.46	3.3764 (15)	163

Symmetry codes: (i) 
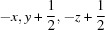
; (ii) 
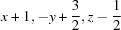
; (iii) 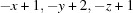; (iv) 
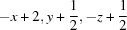
; (v) 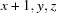.

**Fig. 1.**
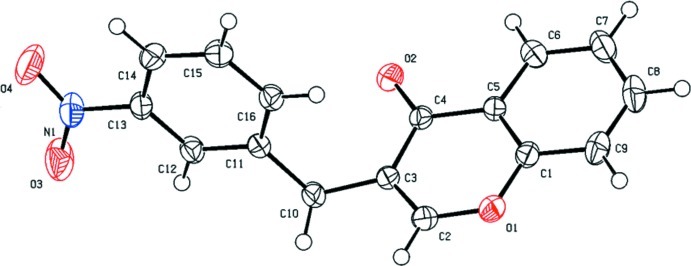
A view of the molecule with displacement ellipsoids drawn at the 50% probability level and H atoms drawn as circles of arbitary size.

## supplementary crystallographic information

### Comment

The title compound, 3-(3-nitrobenzyl)-2-chromen-4-one, belongs to a class of compounds called homoisoflavonoids, which are C-16, *α*,*β* unsaturated carbonyl compounds containing two aromatic rings. Homoisoflavonoids may be categorized into four groups depending on the type of structural backbone present. The four groups are 3-benzyl-4-chromanones, of which the title compound belongs to as well as the, 3-benzylidene-4-chromanones, 3-benzyl-3-hydroxy-4-chromanones and scillascillins (du Toit *et al.*, 2010). The most commonly used procedure for the synthesis of homoisoflavoinoids involves the condensation of chroman-4-one with an aromatic aldehyde in the presence of either an acidic or basic catalyst (Shaikh *et al.*, 2011).

The molecular structure of the title compound is shown in Fig.1. The dihedral angle between the 10-membered co-planar ring O1—C1—C9—C8—C7—C6—C5—C4—C3—C2 and the benzene ring C11—C12—C13—C14—C15—C16 is 77.83 (3)°. In the crystal packing, a number of weak intermolecular C—H···O hydrogen bonds are noted and are listed in Table 1.

### Experimental

A mixture of chroman-4-one (1.00 g, 6.75 mmol), 3-nitrobenzaldehyde (1.25 g, 8.10 mmol) and 10–15 drops of piperidine was heated at 80°C for 10 hrs. The reaction mixture was monitored for completion by thin layer chromatography. Upon completion, the reaction mixture was cooled, diluted with water and neutralized using 10% HCl. To the viscous reaction mixture, 15 ml of ethyl acetate was added. The homoisoflavonoid precipitated out upon the addition of hexane to the reaction mixture. The powdered product was filtered, washed with hexane and dried under vacuum. Upon slow evaporation of chloroform, the crystals of the homoisoflavonoid were obtained with a m.p. of 129–130 °C.

### Refinement

All non-hydrogen atoms were refined anisotropically. All hydrogen atoms were placed in idealized positions and refined with geometrical constraints, and with *U*_iso_(H) = 1.2*U*_eq_(C).

### Crystal data

**Table:**

C_16_H_11_NO_4_	*F*(000) = 584
*M**_r_* = 281.26	*D*_x_ = 1.471 Mg m^−^^3^
Monoclinic, *P*2_1_/*c*	Mo *K*α radiation, λ = 0.71073 Å
*a* = 4.6082 (3) Å	Cell parameters from 16973 reflections
*b* = 10.4219 (6) Å	θ = 1.5–31.6°
*c* = 26.4468 (17) Å	µ = 0.11 mm^−^^1^
β = 90.428 (1)°	*T* = 173 K
*V* = 1270.10 (14) Å^3^	Plate, colourless
*Z* = 4	0.42 × 0.22 × 0.04 mm

### Data collection

**Table:**

Bruker Kappa DUO APEXII diffractometer	4246 independent reflections
Radiation source: fine-focus sealed tube	3069 reflections with *I* > 2σ(*I*)
Graphite monochromator	*R*_int_ = 0.030
0.5° φ scans and ω scans	θ_max_ = 31.6°, θ_min_ = 1.5°
Absorption correction: multi-scan (*SADABS*; Sheldrick, 1997)	*h* = −3→6
*T*_min_ = 0.956, *T*_max_ = 0.996	*k* = −12→15
16973 measured reflections	*l* = −38→36

### Refinement

**Table:**

Refinement on *F*^2^	Primary atom site location: structure-invariant direct methods
Least-squares matrix: full	Secondary atom site location: difference Fourier map
*R*[*F*^2^ > 2σ(*F*^2^)] = 0.047	Hydrogen site location: inferred from neighbouring sites
*wR*(*F*^2^) = 0.138	H-atom parameters constrained
*S* = 1.04	*w* = 1/[σ^2^(*F*_o_^2^) + (0.0728*P*)^2^ + 0.2299*P*] where *P* = (*F*_o_^2^ + 2*F*_c_^2^)/3
4246 reflections	(Δ/σ)_max_ < 0.001
190 parameters	Δρ_max_ = 0.44 e Å^−^^3^
0 restraints	Δρ_min_ = −0.19 e Å^−^^3^

### Special details

**Table:**

Geometry. All e.s.d.'s (except the e.s.d. in the dihedral angle between two l.s. planes) are estimated using the full covariance matrix. The cell e.s.d.'s are taken into account individually in the estimation of e.s.d.'s in distances, angles and torsion angles; correlations between e.s.d.'s in cell parameters are only used when they are defined by crystal symmetry. An approximate (isotropic) treatment of cell e.s.d.'s is used for estimating e.s.d.'s involving l.s. planes.
Refinement. Refinement of *F*^2^ against ALL reflections. The weighted *R*-factor *wR* and goodness of fit *S* are based on *F*^2^, conventional *R*-factors *R* are based on *F*, with *F* set to zero for negative *F*^2^. The threshold expression of *F*^2^ > σ(*F*^2^) is used only for calculating *R*-factors(gt) *etc*. and is not relevant to the choice of reflections for refinement. *R*-factors based on *F*^2^ are statistically about twice as large as those based on *F*, and *R*- factors based on ALL data will be even larger.

### Fractional atomic coordinates and isotropic or equivalent isotropic displacement parameters (Å^2^)

**Table:**

	*x*	*y*	*z*	*U*_iso_*/*U*_eq_	
O1	0.74839 (19)	0.65066 (9)	0.16340 (3)	0.0297 (2)	
O2	0.12234 (19)	0.86867 (9)	0.23054 (3)	0.0307 (2)	
O3	0.0611 (3)	0.69688 (13)	0.46889 (4)	0.0563 (4)	
O4	0.2296 (3)	0.87629 (13)	0.49596 (4)	0.0569 (3)	
N1	0.2075 (3)	0.79369 (12)	0.46362 (4)	0.0364 (3)	
C1	0.6196 (2)	0.74864 (12)	0.13678 (4)	0.0254 (2)	
C2	0.6533 (3)	0.62632 (12)	0.21073 (5)	0.0281 (2)	
H2	0.7393	0.5562	0.2282	0.034*	
C3	0.4471 (3)	0.69311 (11)	0.23514 (4)	0.0243 (2)	
C4	0.3094 (2)	0.80163 (11)	0.21004 (4)	0.0229 (2)	
C5	0.4068 (2)	0.82546 (11)	0.15828 (4)	0.0228 (2)	
C6	0.2875 (3)	0.92474 (12)	0.12884 (5)	0.0304 (3)	
H6	0.1413	0.9783	0.1426	0.037*	
C7	0.3805 (3)	0.94494 (15)	0.08021 (5)	0.0380 (3)	
H7	0.2999	1.0127	0.0606	0.046*	
C8	0.5929 (3)	0.86601 (16)	0.05974 (5)	0.0392 (3)	
H8	0.6559	0.8806	0.0261	0.047*	
C9	0.7132 (3)	0.76728 (14)	0.08729 (5)	0.0343 (3)	
H9	0.8565	0.7131	0.0730	0.041*	
C10	0.3523 (3)	0.65438 (12)	0.28725 (5)	0.0303 (3)	
H10A	0.4365	0.5691	0.2949	0.036*	
H10B	0.1386	0.6446	0.2868	0.036*	
C11	0.4334 (2)	0.74495 (11)	0.32985 (4)	0.0238 (2)	
C12	0.2953 (3)	0.72947 (12)	0.37618 (4)	0.0265 (2)	
H12	0.1554	0.6635	0.3805	0.032*	
C13	0.3633 (3)	0.81075 (12)	0.41575 (4)	0.0261 (2)	
C14	0.5657 (3)	0.90853 (12)	0.41192 (5)	0.0292 (3)	
H14	0.6065	0.9640	0.4396	0.035*	
C15	0.7057 (3)	0.92194 (12)	0.36619 (5)	0.0305 (3)	
H15	0.8482	0.9871	0.3624	0.037*	
C16	0.6411 (3)	0.84133 (12)	0.32557 (5)	0.0265 (2)	
H16	0.7402	0.8523	0.2945	0.032*	

### Atomic displacement parameters (Å^2^)

**Table:**

	*U*^11^	*U*^22^	*U*^33^	*U*^12^	*U*^13^	*U*^23^
O1	0.0335 (4)	0.0285 (4)	0.0271 (4)	0.0059 (3)	0.0036 (3)	−0.0029 (3)
O2	0.0348 (5)	0.0295 (5)	0.0280 (5)	0.0034 (4)	0.0080 (3)	−0.0026 (4)
O3	0.0700 (8)	0.0657 (8)	0.0335 (6)	−0.0301 (6)	0.0160 (5)	−0.0001 (5)
O4	0.0765 (8)	0.0620 (8)	0.0325 (6)	−0.0123 (6)	0.0152 (5)	−0.0182 (5)
N1	0.0398 (6)	0.0462 (7)	0.0233 (5)	−0.0044 (5)	0.0039 (4)	−0.0002 (5)
C1	0.0279 (5)	0.0265 (5)	0.0218 (5)	−0.0018 (4)	0.0010 (4)	−0.0024 (4)
C2	0.0361 (6)	0.0226 (5)	0.0256 (6)	0.0018 (5)	−0.0035 (4)	−0.0009 (4)
C3	0.0329 (6)	0.0202 (5)	0.0198 (5)	−0.0043 (4)	−0.0007 (4)	−0.0008 (4)
C4	0.0267 (5)	0.0210 (5)	0.0212 (5)	−0.0042 (4)	0.0020 (4)	−0.0018 (4)
C5	0.0264 (5)	0.0227 (5)	0.0194 (5)	−0.0023 (4)	0.0010 (4)	−0.0003 (4)
C6	0.0347 (6)	0.0291 (6)	0.0275 (6)	0.0014 (5)	0.0007 (5)	0.0044 (5)
C7	0.0466 (8)	0.0399 (7)	0.0274 (7)	−0.0025 (6)	−0.0010 (5)	0.0105 (6)
C8	0.0419 (7)	0.0533 (9)	0.0224 (6)	−0.0078 (6)	0.0056 (5)	0.0041 (6)
C9	0.0349 (6)	0.0434 (7)	0.0247 (6)	−0.0021 (6)	0.0073 (5)	−0.0057 (5)
C10	0.0455 (7)	0.0238 (5)	0.0215 (6)	−0.0092 (5)	0.0003 (5)	0.0012 (4)
C11	0.0289 (5)	0.0220 (5)	0.0206 (5)	−0.0007 (4)	−0.0010 (4)	0.0018 (4)
C12	0.0298 (5)	0.0269 (5)	0.0230 (6)	−0.0058 (4)	0.0002 (4)	0.0028 (4)
C13	0.0296 (5)	0.0297 (6)	0.0191 (5)	0.0006 (4)	0.0012 (4)	0.0014 (4)
C14	0.0333 (6)	0.0275 (6)	0.0269 (6)	−0.0015 (5)	−0.0029 (5)	−0.0036 (5)
C15	0.0317 (6)	0.0286 (6)	0.0312 (6)	−0.0080 (5)	−0.0001 (5)	−0.0004 (5)
C16	0.0287 (5)	0.0265 (5)	0.0243 (6)	−0.0029 (4)	0.0021 (4)	0.0010 (4)

### Geometric parameters (Å, º)

**Table:**

O1—C2	1.3533 (15)	C7—H7	0.9500
O1—C1	1.3727 (15)	C8—C9	1.375 (2)
O2—C4	1.2377 (14)	C8—H8	0.9500
O3—N1	1.2222 (17)	C9—H9	0.9500
O4—N1	1.2172 (16)	C10—C11	1.5145 (16)
N1—C13	1.4709 (16)	C10—H10A	0.9900
C1—C5	1.3905 (16)	C10—H10B	0.9900
C1—C9	1.3949 (17)	C11—C16	1.3926 (16)
C2—C3	1.3467 (17)	C11—C12	1.3942 (16)
C2—H2	0.9500	C12—C13	1.3804 (17)
C3—C4	1.4546 (16)	C12—H12	0.9500
C3—C10	1.5042 (16)	C13—C14	1.3858 (18)
C4—C5	1.4650 (15)	C14—C15	1.3823 (18)
C5—C6	1.4047 (17)	C14—H14	0.9500
C6—C7	1.3748 (18)	C15—C16	1.3941 (17)
C6—H6	0.9500	C15—H15	0.9500
C7—C8	1.391 (2)	C16—H16	0.9500
			
C2—O1—C1	118.16 (9)	C8—C9—C1	118.37 (12)
O4—N1—O3	123.17 (12)	C8—C9—H9	120.8
O4—N1—C13	118.71 (12)	C1—C9—H9	120.8
O3—N1—C13	118.12 (12)	C3—C10—C11	116.27 (10)
O1—C1—C5	121.47 (10)	C3—C10—H10A	108.2
O1—C1—C9	116.69 (11)	C11—C10—H10A	108.2
C5—C1—C9	121.83 (12)	C3—C10—H10B	108.2
C3—C2—O1	125.53 (11)	C11—C10—H10B	108.2
C3—C2—H2	117.2	H10A—C10—H10B	107.4
O1—C2—H2	117.2	C16—C11—C12	118.25 (11)
C2—C3—C4	119.35 (11)	C16—C11—C10	123.71 (11)
C2—C3—C10	120.76 (11)	C12—C11—C10	118.03 (10)
C4—C3—C10	119.87 (10)	C13—C12—C11	119.51 (11)
O2—C4—C3	122.81 (10)	C13—C12—H12	120.2
O2—C4—C5	122.20 (11)	C11—C12—H12	120.2
C3—C4—C5	114.98 (10)	C12—C13—C14	123.03 (11)
C1—C5—C6	118.15 (11)	C12—C13—N1	117.96 (11)
C1—C5—C4	120.43 (10)	C14—C13—N1	119.00 (11)
C6—C5—C4	121.42 (11)	C15—C14—C13	117.20 (11)
C7—C6—C5	120.52 (12)	C15—C14—H14	121.4
C7—C6—H6	119.7	C13—C14—H14	121.4
C5—C6—H6	119.7	C14—C15—C16	120.98 (11)
C6—C7—C8	119.91 (13)	C14—C15—H15	119.5
C6—C7—H7	120.0	C16—C15—H15	119.5
C8—C7—H7	120.0	C11—C16—C15	121.00 (11)
C9—C8—C7	121.22 (12)	C11—C16—H16	119.5
C9—C8—H8	119.4	C15—C16—H16	119.5
C7—C8—H8	119.4		
			
C2—O1—C1—C5	3.07 (16)	C7—C8—C9—C1	−0.7 (2)
C2—O1—C1—C9	−177.07 (11)	O1—C1—C9—C8	−178.84 (12)
C1—O1—C2—C3	−2.33 (18)	C5—C1—C9—C8	1.02 (19)
O1—C2—C3—C4	−0.18 (19)	C2—C3—C10—C11	111.41 (14)
O1—C2—C3—C10	178.04 (11)	C4—C3—C10—C11	−70.37 (15)
C2—C3—C4—O2	−178.62 (11)	C3—C10—C11—C16	−15.66 (18)
C10—C3—C4—O2	3.14 (17)	C3—C10—C11—C12	165.39 (11)
C2—C3—C4—C5	1.83 (16)	C16—C11—C12—C13	1.26 (17)
C10—C3—C4—C5	−176.41 (10)	C10—C11—C12—C13	−179.73 (11)
O1—C1—C5—C6	179.30 (11)	C11—C12—C13—C14	−0.19 (19)
C9—C1—C5—C6	−0.55 (18)	C11—C12—C13—N1	178.39 (11)
O1—C1—C5—C4	−1.39 (17)	O4—N1—C13—C12	−168.33 (13)
C9—C1—C5—C4	178.76 (11)	O3—N1—C13—C12	11.53 (19)
O2—C4—C5—C1	179.38 (11)	O4—N1—C13—C14	10.31 (19)
C3—C4—C5—C1	−1.07 (15)	O3—N1—C13—C14	−169.83 (13)
O2—C4—C5—C6	−1.33 (18)	C12—C13—C14—C15	−0.96 (19)
C3—C4—C5—C6	178.22 (11)	N1—C13—C14—C15	−179.52 (12)
C1—C5—C6—C7	−0.23 (19)	C13—C14—C15—C16	1.02 (19)
C4—C5—C6—C7	−179.53 (12)	C12—C11—C16—C15	−1.20 (18)
C5—C6—C7—C8	0.5 (2)	C10—C11—C16—C15	179.86 (12)
C6—C7—C8—C9	0.0 (2)	C14—C15—C16—C11	0.0 (2)

### Hydrogen-bond geometry (Å, º)

**Table:**

*D*—H···*A*	*D*—H	H···*A*	*D*···*A*	*D*—H···*A*
C2—H2···O2	0.95	2.32	3.2663 (15)	171
C7—H7···O3^i^	0.95	2.65	3.5614 (19)	160
C8—H8···O3^ii^	0.95	2.54	3.3071 (18)	138
C14—H14···O4^iii^	0.95	2.50	3.4380 (17)	172
C15—H15···O1^iv^	0.95	2.62	3.5569 (15)	170
C16—H16···O2^v^	0.95	2.46	3.3764 (15)	163

Symmetry codes: (i) −*x*, *y*+1/2, −*z*+1/2; (ii) *x*+1, −*y*+3/2, *z*−1/2; (iii) −*x*+1, −*y*+2, −*z*+1; (iv) −*x*+2, *y*+1/2, −*z*+1/2; (v) *x*+1, *y*, *z*.
